# The Cooperation between hMena Overexpression and HER2 Signalling in Breast Cancer

**DOI:** 10.1371/journal.pone.0015852

**Published:** 2010-12-30

**Authors:** Francesca Di Modugno, Marcella Mottolese, Lucia DeMonte, Paola Trono, Michele Balsamo, Andrea Conidi, Elisa Melucci, Irene Terrenato, Francesca Belleudi, Maria Rosaria Torrisi, Massimo Alessio, Angela Santoni, Paola Nisticò

**Affiliations:** 1 Laboratory of Immunology, Regina Elena National Cancer Institute, Rome, Italy; 2 Experimental Chemotherapy, Regina Elena National Cancer Institute, Rome, Italy; 3 Department of Pathology, Regina Elena National Cancer Institute, Rome, Italy; 4 Tumor Immunology, Dibit, San Raffaele Scientific Institute, Milan, Italy; 5 Proteome Biochemistry, Dibit, San Raffaele Scientific Institute, Milan, Italy; 6 Koch Institute for Integrative Cancer Research, Massachusetts Institute of Technology, Cambridge, Massachusetts, United States of America; 7 Department of Molecular and Developmental Genetics, VIB11, Katholieke Universiteit Leuven, Leuven, Belgium; 8 Department of Epidemiology, Regina Elena National Cancer Institute, Rome, Italy; 9 Azienda Ospedaliera S. Andrea, Rome, Italy; 10 Department of Clinical and Molecular Medicine, University ‘Sapienza’, Rome, Italy; Yale Medical School, United States of America

## Abstract

hMena and the epithelial specific isoform hMena^11a^ are actin cytoskeleton regulatory proteins belonging to the Ena/VASP family. EGF treatment of breast cancer cell lines upregulates hMena/hMena^11a^ expression and phosphorylates hMena^11a^, suggesting cross-talk between the ErbB receptor family and hMena/hMena^11a^ in breast cancer. The aim of this study was to determine whether the hMena/hMena^11a^ overexpression cooperates with HER-2 signalling, thereby affecting the HER2 mitogenic activity in breast cancer. In a cohort of breast cancer tissue samples a significant correlation among hMena, HER2 overexpression, the proliferation index (high Ki67), and phosphorylated MAPK and AKT was found and among the molecular subtypes the highest frequency of hMena overexpressing tumors was found in the HER2 subtype. From a clinical viewpoint, concomitant overexpression of HER2 and hMena identifies a subgroup of breast cancer patients showing the worst prognosis, indicating that hMena overexpression adds prognostic information to HER2 overexpressing tumors. To identify a functional link between HER2 and hMena, we show here that HER2 transfection in MCF7 cells increased hMena/hMena^11a^ expression and hMena^11a^ phosphorylation. On the other hand, hMena/hMena^11a^ knock-down reduced HER3, AKT and p44/42 MAPK phosphorylation and inhibited the EGF and NRG1-dependent HER2 phosphorylation and cell proliferation. Of functional significance, hMena/hMena^11a^ knock-down reduced the mitogenic activity of EGF and NRG1. Collectively these data provide new insights into the relevance of hMena and hMena^11a^ as downstream effectors of the ErbB receptor family which may represent a novel prognostic indicator in breast cancer progression, helping to stratify patients.

## Introduction

Breast cancer is a heterogeneous disease and in recent years the introduction of new targeted therapeutic approaches highlights the need to stratify patients and consequently to identify accurate biomarkers to select the best therapeutic choice. The molecular classification of breast tumors has identified tumor subtypes, among which the overexpression of the ErbB family of receptors is confined to tumors with unfavourable prognosis [1]. The human ErbB receptor family comprises four tyrosine kinases members (EGFR, HER2, HER3 and HER4) and their deregulation has been correlated with cancer development and progression [1], [2]. Upon ligand binding ErbB receptors undergo homodimerization or heterodimerization and activate a complex signalling network that controls tumor cell proliferation as well as motility through different pathways that regulate rearrangements of the actin cytoskeleton [3]. HER2 functions as a common co-receptor recruited from EGFR or HER3 upon binding to their ligands [4] and HER2/HER3 heterodimer has been defined as the major oncogenic unit in HER2 positive breast cancer [5], [6]. The two main signalling pathways downstream from the ErbB receptors are the phosphatidylinositol 3′-kinase (PI3K) and the mitogen activated protein kinase (MAPK) [4]. PI3K activation results in the phosphorylation of PIP2 to yield PIP3 which in turn activates several downstream signalling molecules including AKT and regulates actin regulatory proteins such as cofillin and capping proteins [7], [8]. MAPK activation is responsible for the EGF dependent mitogenic effect in normal and transformed mammary epithelia [9] and its role in cell cycle progression is also sustained by cytoskeletal organization [10].

hMena (ENAH), the human ortholog of murine Mena [11], is a member of the Ena/VASP protein family which in mammalians includes Mena, VASP and Evl. Ena/VASP are key actin regulatory molecules that control cell shape, adhesion and migration [12], [13], processes that are frequently deregulated following neoplastic transformation. Ena/VASP proteins behave as homo and heterotetramers, and the functions of the three members are redundant in cells and *in vivo*. However, only Mena may be regulated by the tumor suppressor Tes, which binds the EVH1 domain of Mena and not VASP or EVL through an unconventional Lim-domain mediated interaction [14].

Recently, we have demonstrated that hMena overexpression represents an early marker of breast tumorigenesis, being undetectable in normal breast epithelium, and overexpressed in benign breast lesions with an increased risk of transformation and, in more than 70% of tumors, with HER2+, ER/PgR- and high Ki67 phenotype [15]. While Neuregulin-1 (NRG1) and EGF growth factors induce an increase of hMena expression, Herceptin treatment down-regulates hMena in breast cancer cell lines overexpressing HER2 [15], [16]. The hMena isoform hMena^11a^, recently characterized by our group, shows an additional 21 amino acids betweeen the F-actin binding and the coiled-coild domain in the EVH2 domain which possesses three putative phosphorylation sites [16]. This isoform undergoes phosphorylation upon long term stimulation with EGF, associated with p44/42 MAPK activation and with an increased proliferation rate in breast cancer cell lines [16]. hMena^11a^ expression is associated with an epithelial phenotype [16], [17], [18] and identifies human pancreatic adenocarcinoma cell lines that are sensitive to the EGFR inhibitor Erlotinib [17]. Thus, we hypothesize that cross-talk takes place between the ErbB receptor family and hMena.

In the present work, through a combination of experimental and *in vivo* studies, we have demonstrated that hMena/hMena^11a^ are downstream targets of HER2 activity and that they affect HER2 signalling. Moreover, hMena overexpression is a frequent event in the HER2 breast tumor subtype and significantly correlates with HER2 overexpressing tumors and an activated status of MAPK and AKT. On the contrary, depletion of hMena by specific RNA interference reduces the phosphorylation of HER3, p44/42 MAPK and AKT and inhibits both EGF and NRG1-mediated phosphorylation of HER2 and EGFR as well as cell proliferation. Of note in breast cancer patients, undetectable levels of hMena are more frequent in luminal breast tumors, and discriminate HER2 overexpressing tumors with downstream inactive pathways carried by patients that have a better prognosis. Conversely, the concomitant overexpression of HER2 and hMena identifies a subgroup of breast cancer patients with a worse prognosis indicating that hMena overexpression adds prognostic information in HER2 overexpressing tumors.

## Results

### hMena overexpression occurs more frequently in the HER2 subtype and correlates with Ki67, MAPK and AKT activity

**Figure 1:**
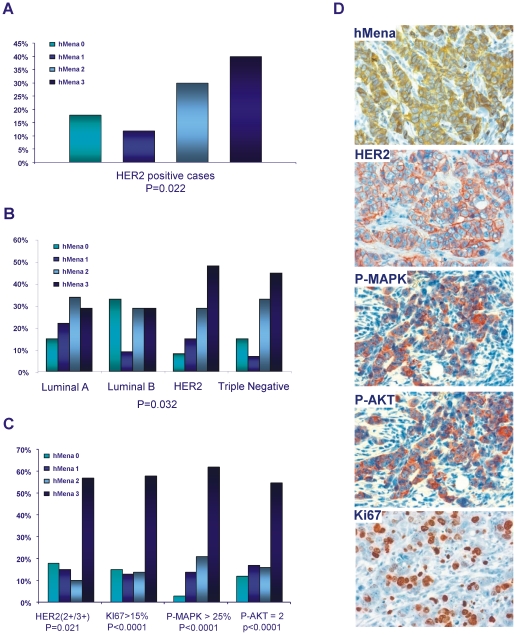
hMena is differently distributed among breast cancer subtypes and correlates with HER2 overexpression, MAPK, AKT activity and Ki67. *A.* Relationship between hMena staining and HER2 positivity in human breast carcinomas. Histograms represent the percentage of the hMena staining (score 0; 1; 2; 3) in 82 HER2 positive breast cancer samples (2+ with gene amplification and 3+) (p = 0.02). *B.* hMena distribution within 286 human breast carcinomas grouped according to molecular subtypes. hMena expression (0, 1, 2, 3 scores) in Luminal A, Luminal B, HER2 subtypes and Triple Negative. The percentage of hMena-positive cases (score 3) is significantly higher in the HER2 subtype and Triple-Negative in comparison to Luminal A and Luminal B breast cancer (p = 0.032). *C.* Correlation between hMena expression and HER2, P-AKT, P-MAPK and Ki67 in a subgroup of 178 breast cancer patients. Percentage of HER2 positivity, high Ki67 proliferation index, elevated P-AKT and P-MAPK expression for each hMena score. The percentage of HER2 positivity (p = 0.016), P-AKT (p<0.001) and P-MAPK (p<0.001) overexpression, high Ki67 proliferation index (p = 0.016) increase significantly in relationship to hMena score (0 to 1 to 2 to 3). *D*. Representative case of an invasive ductal carcinoma with a strong hMena positivity (score 3+) displaying HER2 overexpression (score 3 by IHC), P-MAPK and P-AKT positivity, elevated Ki67 index. Magnification, 40x. Original scale 1 cm = 30micron (25 cm×33 cm).

We have evaluated hMena expression in a series of 286 primary breast cancer (BC) classified in Luminal A (ERα/PR+ HER2-), Luminal B (ERα/PR+ HER2+), Triple Negative (ERα/PR/HER2−) and HER2 subtype (ERα/PR− HER2+), as indicated in Table 1. The immunohistochemical analysis demonstrated a significant linear trend (p = 0.022) between hMena and HER2 expression, independent of breast tumor subtype. Figure 1 panel A shows the frequency of HER2 positive tumors (2+ with gene amplification and 3+ score) with different hMena staining. Of interest, hMena is differently distributed within the four BC subtypes (p = 0.032). In fact, only 29% of the Luminal A and Luminal B subtypes overexpressed hMena with a score 3, whereas 48% and 44% of the HER2 and the Triple Negative subtypes, respectively showed hMena overexpression (Figure 1 panel B).

**Table 1:** Clinico-pathological characteristics of the patients.

CHARACTERISTICS	No of cases	%
**Number of patients**	**286**	
**Median age, y (range)** 53 (28–86)		
**hMena**		
Negative (score 0/1)	96	34
Positive (score 2/3)	190	66
**Histotype**		
Invasive ductal carcinoma	246	86
Invasive lobular carcinoma	28	10
Other carcinomas	12	4
**Histologic grade**		
Grade 1	27	10
Grade 2	146	54
Grade 3	96	36
**Lymph node status**		
Negative	154	54
Positive	132	46
**Tumor size**		
T1	171	60
T2	80	29
T3,4	28	11
**ER**		
Negative	89	31
Positive	197	69
**PgR**		
Negative	107	37
Positive	179	63
**Ki67**		
Negative (≤15)	164	57
Positive (>15)	122	43
**HER-2**		
Negative	204	71
Positive	82	29
**Molecular Subtypes**		
Luminal A	177	62
Luminal B	34	12
Triple Negative	27	9
HER2 subtype	48	17

To analyze downstream signalling pathways of the ErbB family by immunohistochemistry (IHC), the expression of the phospho-MAPK (P-MAPK) and phospho-AKT (P-AKT) were analyzed in 178 BC randomly selected from the entire series of 286 patients included in our study. The IHC findings, reported in Figure 1 panel C, clearly demonstrate a significant correlation between hMena (score 3), HER2 overexpression (p = 0.021) and the proliferation index as assessed by Ki-67 (p<0.0001), as we have already reported in a different cohort of patients [15]. A strong correlation among hMena overexpression and P-AKT and P-MAPK positivity (p<0.0001 in both cases) was found. Immunohistochemical analyses of serial sections of a representative invasive BC are reported in Figure 1 panel D.

**Figure 2:**
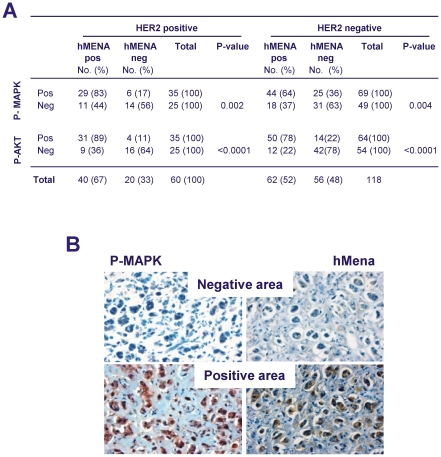
hMena overexpression correlates with the phosphorylated status of MAPK and AKT. ***A.*** The table evidences that in 60 HER2 positive and 118 HER2 negative breast tumors the percentage of P-MAPK and P-AKT positive breast tumors is significantly higher in hMena positive (score 2/3) than in hMena negative breast tumors (score 0/1). ***B.*** A representative, although not a paradigmatic case, where P-MAPK displays a heterogeneous immunostaining (40% tumor cells). P-MAPK positivity is mostly confined in the tumor area (positive area) concomitantly showing a strong hMena (score 3) immunoreactivity.

To evaluate the role of hMena overexpression in MAPK and AKT activation, we categorized in hMena negative (score 0/1) and hMena positive (score 2/3) 60 HER2 positive and 118 HER2 negative primary BC of 178 cases analyzed. As reported in Figure 2 panel A, only 17% and 11% HER2-positive/hMena-negative tumors expressed P-MAPK and P-AKT, whereas the majority of HER2-positive/hMena-positive tumors showed an activated status of both MAPK (83.1%) and AKT (89%). In HER2 negative tumors hMena expression has a minor contribution in MAPK and AKT activity, suggesting that hMena overexpression cooperates with HER2 in sustaining MAPK and AKT activity. Interestingly, as shown in a representative, although not paradigmatic case (Figure 2 panel B) in which P-MAPK displayed a heterogeneous immunostaining (40% tumor cells), hMena positivity was mainly found in the tumor area positive for P-MAPK and viceversa.

### HER2 overexpression and activity affect hMena/hMena^11a^ overexpression in human breast cancer cell lines

**Figure 3:**
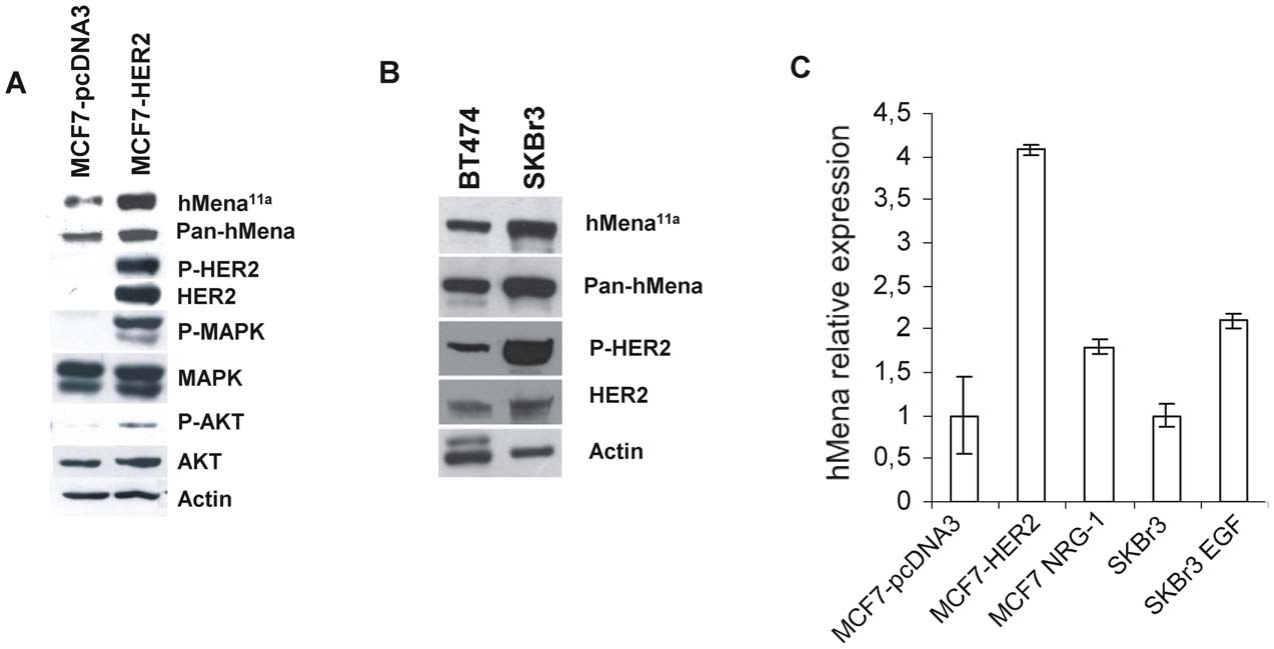
HER2 overexpression and activity affect hMena/hMena^11a^ expression in human breast cancer cell lines. *A.* HER2 transfection affects hMena expression in MCF7 breast cancer cells. Western blot analysis of MCF7-pcDNA3 and MCF7-HER2 lysates (50 µg) with the indicated antibodies. Two different antibodies for hMena have been used: the pan-hMena antibody, recognizing hMena and hMena^11a^ as a doublet of 88–90 kDa, and the anti-hMena^11a^ specific antibody. Membranes were sequentially stripped and reprobed with the indicated total and phospho-specific antibodies. *B.* hMena/hMena^11a^ expression in HER2 overexpressing breast cancer cell lines. Western blot analysis of BT474 and SKBr3 breast cancer cell line lysates (50 µg) with the indicated antibodies. As loading control, blots were probed with anti-Actin mAb (1 µg/ml). *C.* HER2 transfection, NRG and EGF treatment affect hMena expression at mRNA level. QRT-PCR analysis of hMena and housekeeping β-Actin mRNA levels performed on 10 ng of total RNA from the untreated MCF7-pcDNA3, HER2 transfected MCF7, or NRG1 treated MCF7 breast cancer cell line and SKBr3 cells either untreated or treated with EGF. Results are given as the ratio between *hMena* and *β-Actin* genes relative to the internal control.

We previously reported that epithelial cancer cell lines express hMena and the epithelial specific hMena^11a^ isoform [16]. To investigate the functional link between hMena/hMena^11a^ and HER2, we stably transfected HER2 in MCF7, a luminal epithelial breast cancer cell line with low HER2 endogenous levels [19]. HER2 overexpression in MCF7 cells leads to the upregulation of hMena and hMena^11a^ compared to control cells (MCF7-pCDNA3) as evaluated by pan-hMena and specific hMena^11a^ antibodies (Figure 3 panel A). This was associated with a phosphorylation of the tyrosine Y1248 in HER2 receptor and the activation of MAPK and PI3K as documented by the amount of phosphorylated p44/42 MAPK and AKT (Figure 3 panel A). Phosphorylation at Y1248 of HER2 was also observed in BT474 and SKBr3 BC cells, naturally overexpressing HER2 and hMena/hMena^11a^ (Figure 3 panel B). Real-time qRT-PCR experiments demonstrated a 4 fold upregulation of hMena mRNA in MCF7-HER2 cells with respect to MCF7-pcDNA3 control cells. Treatment for 24 h of MCF7-pcDNA3 and SKBr3 cells respectively with NRG1 (10 ng/ml) and EGF (100 ng/ml), factors that indirectly activate HER2 by heterodimerization with HER3 and EGFR respectively, determined an increase in hMena RNA (1.8 and 2.1 fold respectively) (Figure 3 panel C). These results show that the growth factor-mediated hMena protein upregulation reported previously [15], [16] is related to an increase in hMena mRNA and indicate that HER2 overexpression and activity contribute to hMena/hMena^11a^ overexpression in BC cell lines.

### HER2 overexpression affects hMena^11a^ phosphorylation in breast cancer cells

**Figure 4:**
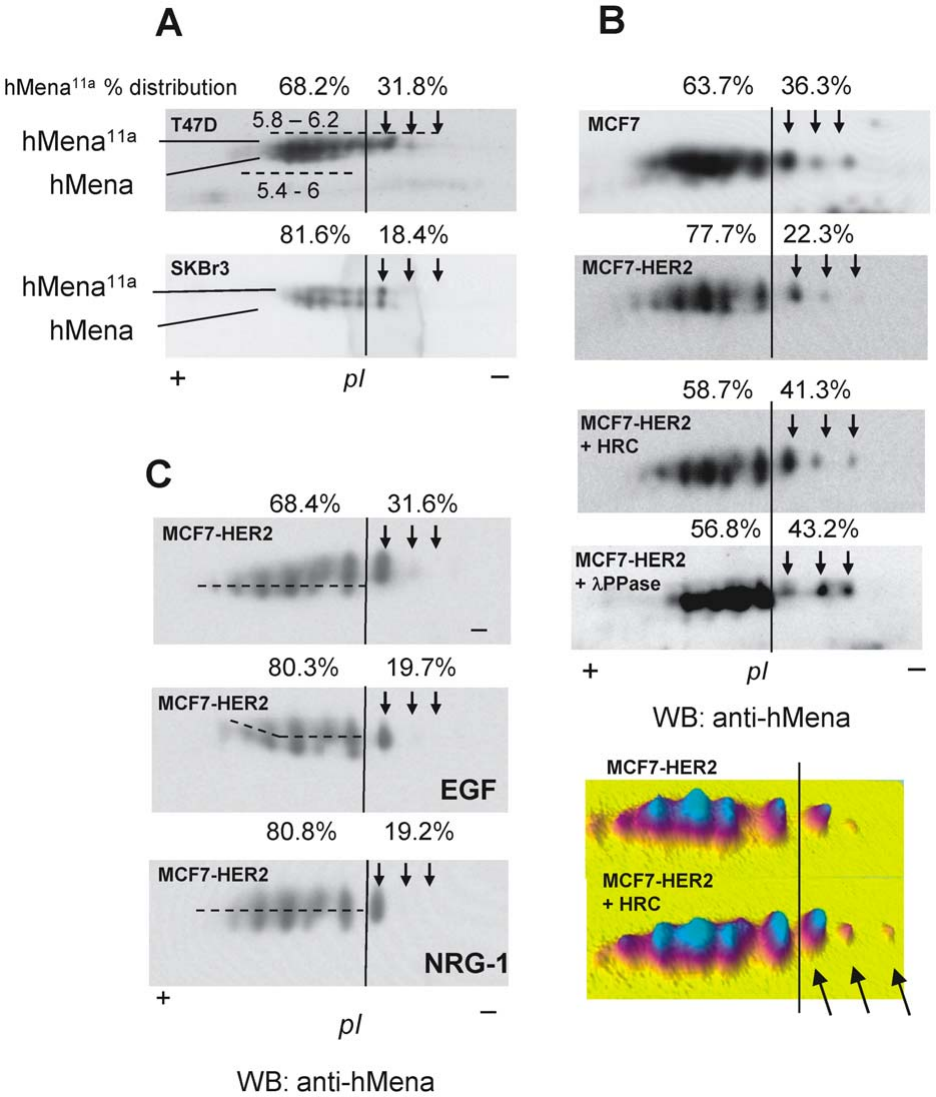
HER2 overexpression affects hMena^11a^ phosphorylation in breast cancer cells. ***A.*** Proteins from T47D and SKBr3 cell lines were resolved by 2D-electrophoresis on a pH 3–10 nonlinear range and 7.5% acrylamide SDS-PAGE. Proteins were then electrontransferred to nitrocellulose and hMena reactivity was revealed by Western blot. Protein spot trains were compared by using Progenesis PG240 v2006 software (Nonlinear dynamics, Newcastle, UK). The set of spots corresponding to hMena and hMena^11a^ are indicated including the isoelectric point range (dashed line). The phosphorylation threshold (arrows and vertical bars) has been arbitrarily defined as described [13]. ***B.*** Lysates of MCF7 cells, either stably transfected with HER2 or with empty vector (pcDNA3) were resolved by 2D-electrophoresis as described above, showing that a higher fraction of hMena^11a^ is constitutively phosphorylated in MCF7-HER2 cells. MCF7-HER2 cells treated with Herceptin for 48 h (MCF7-HER2+ HRC) show a fraction of phosphorylated hMena^11a^ similar to the dephosphorylated MCF7-HER2 lysates. MCF7-HER2 cell lysates were also incubated without or with λ-phosphatase (λ-PPase) before 2D analysis. Arrows indicated hMena^11a^ spots after phosphatase treatment. Below the densitometric analysis of the hMena spots revealed by Western blot in MCF7-HER2 cells treated without (- HRC) or with Herceptin for 48 h (+ HRC) are shown. Arrows indicated hMena^11a^ spots that, after Herceptin treatment, resulted to be dephosphorylated. Pseudo-colours three-dimensional view has been obtained by using Progenesis PG240 v2006 software. ***C.*** Proteins from the MCF7-HER2 cell lines incubated with or without 100 ng/ml EGF or 10 ng/ml NRG1 for 24 h in serum starved medium, were resolved by 2D-electrophoresis and analyzed as described in panel A.

EGF treatment of BC cell lines promotes concomitant up-regulation of hMena and hMena^11a^, resulting in a higher fraction of phosphorylated hMena^11a^ isoform. Moreover, hMena^11a^ overexpression and phosphorylation lead to an increase in p44/42 mitogen-activated protein kinase (MAPK) activation and cell proliferation [16]. Thus we analyzed whether hMena^11a^ phosphorylation is downstream to HER2 signalling. T47D and MCF7 cells, with low endogenous levels of HER2 [19], display only modest hMena^11a^ phosphorylation as evaluated by 2D-E analysis (Figure 4, panels A and B). Conversely, SKBr3 cells, endogenously overexpressing highly phosphorylated HER2 (Figure 3 panel B) show high amounts of phosphorylated hMena^11a^ (Figure 4 panel A). To better define hMena^11a^ as a downstream target of HER2, the levels of hMena^11a^ phosphorylation were evaluated in MCF7-HER2 cells. As depicted by the pattern of spots presented in Figure 4 panel B, a higher percentage (77.7% *vs.* 63.7%) of phosphorylated hMena^11a^ was seen in the MCF7-HER2 with respect to control (MCF7) cells, as revealed by the more pronounced shift toward lower pH, which is significantly reduced by λ phosphatase treatment. Herceptin treatment down-regulates hMena in MCF7-HER2 cells (data not shown), confirming our previous results in endogenously HER2 overexpressing BC cell lines [15]. Thus, to determine if Herceptin treatment is able to inhibit HER2-dependent hMena^11a^ phosphorylation, MCF7-HER2 cells were treated with Herceptin 25 µg/ml for 48 h, and the percentage of phosphorylated hMena^11a^ was reduced (58.7% vs 77.7%) to a level similar to that observed after λ phosphatase treatment (Figure 4 panel B).

To evaluate whether hMena^11a^ phosphorylation is further increased by ligand dependent HER2 activation, we treated MCF7-HER2 cells either with EGF or NRG1. After 24 h of EGF treatment, a higher amount of hMena^11a^ was phosphorylated (80.2%) compared to untreated starved cells (68.3%) (Figure 4 panel C), suggesting that paracrine signals may affect hMena^11a^ phosphorylation. NRG1 treatment induced a phosphorylation pattern of hMena^11a^ (80.8%), similar to that observed with EGF treatment. Overall, these results show that hMena^11a^ is downstream to either the EGFR/HER2 or HER2/HER3 signal transduction pathways. These findings were confirmed in the spontaneously HER2 overexpressing SKBr3 cells where treatment with both EGF and NRG1 slightly increased the phosphorylation level of the constitutively high phosphorylated hMena^11a^ (data not shown).

### hMena knock-down inhibits ErbB family signalling and the EGF/NRG1-mediated mitogenic effect in MCF7-HER2 cells

**Figure 5:**
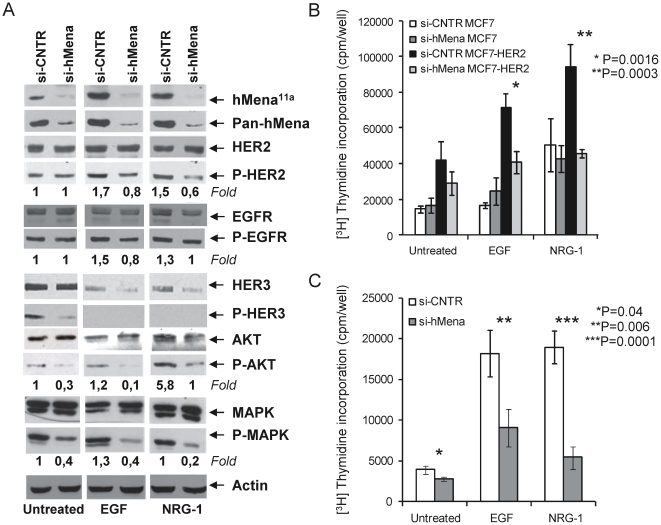
hMena knock-down affects HER2 signalling and inhibits the EGF/NRG1-mediated mitogenic effects in MCF7-HER2 cells. ***A.*** Western blot analysis of MCF7-HER2 breast cancer cell line after 72 h transfection with control and hMena/hMena^11a^ -specific siRNAs, untreated or treated with EGF (100 ng/ml) or NRG1 (10 ng/ml) for the last 24 h of transfection. hMena/hMena^11a^ expression (evaluated by pan-hMena and hMena^11a^ specific antibodies) and phosphorylation status of HER2, EGFR, HER3, AKT and MAPK (p44/42) from whole cell lysates were assessed. Membranes were sequentially stripped and reprobed with the indicated total and phospho-specific antibodies. Densitometric quantitation of anti-P-HER2, anti-P-AKT and anti-P-MAPK immunoreactivity was determined by Quantity One software (Biorad) and normalized in comparison with the Actin immunoreactivity. Densitometric quantitation of anti-P-HER3 immunoreactivity was not determined due to the fact that the total HER3 expression level is not unchanged following treatments. ***B–C.*** Silencing of hMena/hMena^11a^ reduces EGF and NRG1-mediated cell proliferation of HER2 overexpressing MCF7-HER2 (B) and MDA-MB-361 (C) cell lines, but has no significant effect in MCF7 cells (B). Proliferation assays were conducted 72 h after the siRNA transfection by measuring [3H]thymidine incorporation as described in Materials and Methods. Si-CNTR: MCF7, MCF7-HER2 and MDA-MB-361 cells transfected with non-targeting siRNA; Si-hMena: MCF7, MCF7-HER2 and MDA-MB-361 cells transfected with specific hMena/hMena^11a^ siRNA. Histograms represent the mean of three different experiments. Bars, Standard deviations. *P*, according to Student's *t* test (two tailed).

To investigate whether hMena/hMena^11a^ overexpression could affect HER2 expression and activation, the MCF7-HER2 cells were depleted for hMena/hMena^11a^ by siRNA. hMena silencing in starved cells did not affect HER2 and EGFR expression and phosphorylation, but was associated with a reduction in phospho-HER3, phospho-AKT and phospho-p44/42 MAPK levels (Figure 5 panel A). On the other hand, hMena/hMena^11a^ knock-down prevented EGF and NRG1 mediated HER2 and EGFR activation. In particular, hMena/hMena^11a^ depleted cells (si-hMena) treated with EGF or NRG1 for 24 h showed a reduction of phospho-HER2 and phospho-EGFR levels with respect to control cells (Si-CNTR) (Figure 5 panel A). Phospho-HER3 was not detectable following EGF and NRG1 treatment, due to treatment induced down-regulation of the HER3 protein, more evident in the hMena/hMena^11a^ depleted cells.

To explore the hypothesis that hMena/hMena^11a^ might have a functional role in EGF and NRG1 mediated mitogenic effects, ^3^H-thymidine incorporation assays were performed. hMena/hMena^11a^ knock-down induced a slight decrease in the proliferation rate of the silenced MCF7-HER2 cells with respect to the control in untreated conditions and abolished the proliferation activity mediated by EGF and NRG1 (Figure 5 panel B). Similar results were obtained in MDA-MB-361 cells naturally expressing high levels of HER2 and HER3 (Figure 5 panel C and Figure S1). On the contrary, in MCF7, cells with a low HER2 level, hMena/hMena^11a^ knock-down (Figure S1) has no significant effect on the proliferation rate in untreated conditions or in EGF treated cells. A slight effect was observed in NRG1-mediated proliferation (Figure 5 panel B).

### hMena overexpression adds prognostic information in BC patients with HER2 overexpressing tumors

In view of the biochemical and functional data obtained, the prognostic impact of hMena overexpression on disease free survival (DFS) was further estimated in our series of 286 BC patients with a median follow-up of 45 months (1–188 months) in which 88 relapses were recorded.

**Figure 6:**
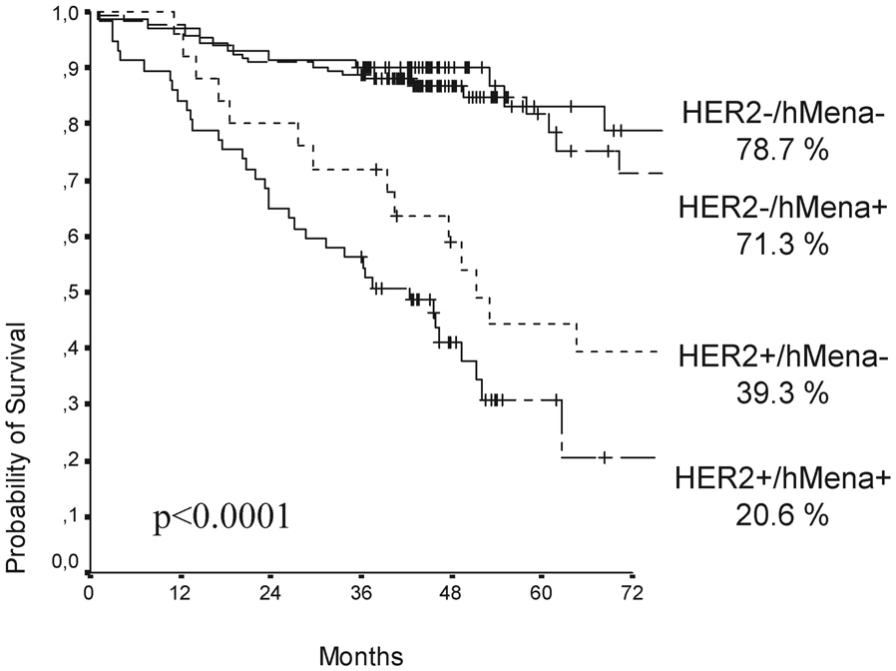
hMena overexpression adds prognostic information in HER2 overexpressing tumors. Kaplan-Meier estimates of disease-free survival (DFS) for hMena and HER2 status show that hMena/HER2 covariates have a different effect on DFS in relationship to the different phenotype combinations. In particular, the lower percentage of disease free patients at 6 year follow up was found in the group of HER2+/hMena+ tumors (p<0.0001).

When the whole series was analyzed by unadjusted Kaplan-Meier curves, hMena/HER2 covariates had different effect on DFS (Figure 6). In particular, in HER2 negative cases hMena positivity slightly reduced the DFS (78.7% BC patients bearing HER2-/hMena- primary tumor and 71.3% with HER2-/hMena+ tumors were disease free after 6 years follow up). Differently, among the HER2 positive cases, only 20.6% of the patients were disease free at a 6 year of follow-up when hMena was overexpressed with respect to 39.3% of patients with HER2+/hMena- tumors (p<0.0001). These data indicate that hMena overexpression adds prognostic information in breast cancer patients with HER2 overexpressing tumors.

## Discussion

Previous experimental and *in vivo* data from our group suggested that the cytoskeleton regulatory protein hMena and its splice-variant deriving isoform, hMena^11a^, may couple ErbB family signalling to the actin cytoskeleton leading to cell proliferation in breast cancer [16]. HER2 positive breast cancers vary greatly in prognosis and response to therapy, underlying the need to identify new markers to further stratify HER2 positive tumors [20]. Here we present our attempts to provide clinical and experimental evidence that hMena/hMena^11a^ are downstream targets and effectors of HER2 activity. The clinical data show that a link exists between hMena and HER2 overexpression, confirming our previous results [15], [16] and further evidences that, according to the molecular classification of breast tumors [21], hMena overexpression is more frequent in the HER2 subtype. On the other hand, the finding that hMena overexpression is correlated with the expression of phosphorylated MAPK and AKT and a high proliferation index in a cohort of primary breast tumors suggests a role for hMena in sustaining an activated status of HER2 pathways. Thus we attempted to delineate the mechanisms linking HER2 activity with hMena in breast cancer cell lines. Exogeneous overexpression of HER2 or stimulation by EGF and NRG1 of different breast cancer cell lines induce the up-regulation of hMena both at the mRNA and protein levels, which suggests a link between ErbB family activation and hMena. Apart from the hMena overexpression, HER2 activation promotes the phosphorylation of the epithelial specific hMena^11a^ isoform [16]. This is indicated by the higher percentage of phosphorylated hMena^11a^ observed in the MCF7-HER2 cells with respect to control and confirmed by the hMena^11a^ dephosphorylation in the same cells treated with Herceptin. Although further studies are needed to clarify the role of hMena^11a^ phosphorylation, we hypothesize it may alter the regulation of actin dynamics, since the 11a peptide is located in the EVH2 domain, the site of protein tetramerization and binding to the grown ends of actin filaments. Studies are in progress in order to define the residue and the kinase involved in phosphorylation. A recent work has described Evl-I, a splice variant derived isoform of Evl, that similarly to hMena^11a^ shows a 21aa peptide included in the EVH2 domain which is phosphorylated by PKD1 and regulates lamellipodia formation and membrane ruffling [22]. In view of the ability of Ena/VASP proteins to form functional tetramers [23], we can hypohesize that the phosphorylation of the two members of Ena/VASP family may implicate a similar function, and a similar regulatory pathway. In line with our results on hMena^11a^ phosphorylation, Janssens K and coauthors [22] report that Evl-I is strongly phosphorylated in the HER2 overexpressing SKBr3 breast cancer cell line, whereas a lower level of P-Evl-I expression is shown in MCF7 and T47D cells.

Growth factor treatment further increased the hMena^11a^ phosphorylation in HER2 overexpressing cancer cells, suggesting that stroma mediated paracrine mechanisms not only induce hMena overexpression, but also sustain the phosphorylation of hMena^11a^. Members of HER family take part in a complex array of combinatorial interactions through the formation of homo and heterodimers between the different family members and not only the preferred heterodimerization partner HER2, but also EGFR and recently HER3 have been defined as important therapeutic targets in breast cancer [6]. Our data demonstrating that hMena/hMena^11a^ expression as well as hMena^11a^ phosphorylation are induced by EGF and NRG1, indicate that hMena/hMena^11a^ are downstream to different receptor complexes (EGFR/HER2 and HER2/HER3), and might thus represent a relevant target for therapeutic regulation.

Functionally, we suggest a dynamic reciprocal cross-talk between ErbB family signalling and actin cytoskeleton regulators, as pointed out by the results that hMena/hMena^11a^ knock-down reduces the phosphorylation of HER3, MAPK and AKT and abolishes the EGF and NRG1 mediated phosphorylation of HER2 and EGFR. The HER2/HER3 receptor pair form the most potent mitogenic receptor complex and HER2 overexpressing tumors frequently show phosphorylated HER3 [24]. Consistently, in our model the HER2 overexpressing MCF7 cells showed a ligand independent phosphorylated status of HER3 receptor. One could envisage that hMena/hMena^11a^ are involved in the heterodimerization of HER2/HER3 as suggested by the reduction of HER3 phosphorylation observed in hMena/hMena^11a^ knock down cells. This could also be the reason for the reduction of phosphorylated AKT and MAPK in untreated cells, since the HER2/HER3 complex is a potent activator of PI3K and MAPK signalling [25]. As already reported, growth factor stimulation determines a down-regulation of HER3 caused by the ligand-stimulated degradation of the receptor, that does not involve HER2 despite the dimerization of the two receptors [26]. This reduction at the protein level is more evident in hMena/hMena^11a^ silenced cells, suggesting a role of hMena/hMena^11a^ also in the post-transcriptional mechanisms regulating the HER3 receptor. The hypothesis that hMena/hMena^11a^ play a role in the heterodimerization and activation of the ErbB family of receptors, an event sustained by actin cytoskeleton organization [27], is reinforced by the reduction of ligand-dependent phosphorylation of HER2 and EGFR in the hMena/hMena^11a^ knock down cells.

hMena/hMena^11a^ knock-down is accompanied by the inhibition of cell proliferation in HER2 overexpressing cells, inhibition which become significant in EGF and NRG1 treated cells, thus supporting the hypothesis that hMena/hMena^11a^ are relevant mediators of the EGF and NRG1 mitogenic signals. Data presented here indicate the ability of hMena/hMena^11a^ to sustain EGF and NRG1-mediated signalling responsible for a proliferative signature of breast cancer. The ErbB family is at the center of converging signals for cell proliferation and motility and it could be hypothesized that the regulation of hMena alternative splicing could contribute to this different cell behaviour. In line with this hypothesis, the recently described murine invasion isoform, Mena INV, sensitizes rat mammary tumor cells to EGF-dependent invasion and protrusion [28]. Moreover, in invasive tumor cells the 11a isoform is down-modulated with respect to the stationary ones [29], according with our previous data on the exclusive expression of hMena^11a^ isoform in epithelial non invasive breast and pancreatic tumor cell lines [16], [17] and with the recent finding reporting the hMena^11a^ expression under the control of epithelial specific splicing regulators [18]. This suggests that the availability of specific hMena isoform antibodies may represent a new tool in the clinical management of breast cancer.

The clinical data presented herein indicate that hMena cooperates with HER2 overexpression in breast cancer progression. In fact, HER2 overexpressing tumors lacking hMena overexpression frequently showed inactivated PI3K and MAPK pathways and the patients have a better prognosis with respect to those co-overexpressing HER2 and hMena. Overexpression of HER2 in the primary tumor represents one of the best prognostic indicators of breast cancer progression [30]. HER2 positive tumors were found to have elevated levels of phosphorylated HER3 [24], suggesting that the recruitment of HER3 contributes to malignant growth. On the other hand, co-overexpression of multiple ErbB family members is associated with decreased survival [31]. Although future studies are needed to clarify the role of hMena and its isoforms in breast cancer progression, our observation raised the possibility that hMena overexpression may contribute in mediating the activation of various ErbB receptors. As the inactivation of ErbB receptors is a new tool in cancer treatment, future studies could lead to the development of hMena overexpression as a marker of breast cancer progression.

## Materials and Methods

### Cell lines

The human breast carcinoma cell lines MCF7, BT474, SKBr3, MDA-MB-361, T47D and BT549 purchased from the ATCC (Rockville MD) were cultured in RPMI 1640 (Gibco, Invitrogen, Pisley, UK) supplemented with 10% fetal calf serum, glutamine, at 37°C in 5% CO_2_-95% air. The DAL cell line was obtained from the ascitic fluid of a breast cancer patient [32]. MCF7-HER2 and MCF7-pcDNA3 stable transfectants were obtained by selecting MCF7 cells transfected with HER2-pcDNA3.1 (kindly provided by Dr. Oreste Segatto), and with the empty vector respectively, using G418 500 µg/ml (Invitrogen) in complete culture medium. BT549 and DAL stable transfectants (shown in Figure S2) were obtained by selecting transfected hMena^11a^-pcDNA3.1 cells with G418. All cell lines were routinely morphologically checked by microscope, growth curve analysis by 3H-Thymidine incorporation assay and Mycoplasma detection (Roche, Monza, Italy).

### Cell treatments

Cells were grown in 6 well plates to 50% confluence in RPMI supplemented with 10% fetal bovine serum. Eighteen hours after medium replacement with fresh medium containing 0.5% serum, cells were treated with recombinant human EGF 100 ng/ml (Promega Corporation, Madison, WI, USA), or NRG1 10 ng/ml (Promega Corporation) for 24 h.

Herceptin (Roche, Monza, Italy) for clinical and *in vitro* use was stored at 4°C and adjusted to the final concentration of 25 µg/ml with culture medium containing 10% serum. Exponentially growing cells were exposed to the treatment for 48 h. Treated and control cells were washed and processed according to the experiment to be done.

### Western blot analysis

Cells were lysed for 30 min at 4°C in 10% glycerol, 0.1% SDS, 0.5% DOC, 1% NP-40 in PBS containing protease and phosphatase-inhibitors. Lysates were centrifuged and protein quantification of supernatants was determined using BCA Protein Assay Reagent (Pierce, Rockford, IL, USA). Lysates (50 µg) were resolved on 10% polyacrylamide gel and transferred to nitrocellulose membrane (GE-Healthcare, Little Chalfont, UK). For HER3 and Phospho-HER3 detection, 80 µg of lysates were used. Blots were blocked for 1 h with 3% skimmed milk in TBST and probed in 3% skimmed milk/TBST overnight at 4°C with the following antibodies: 10 µg/ml anti-hMena rabbit CKLK1 (pan-hMena) antibody [15]; 1 µg/ml anti-hMena^11a^ rabbit antibody, that we developed against a portion of the peptide 11a (RDSPRKNQIVFDNRSYDS) by the use of the Primm service (Primm srl, Milan, Italy), showing a similar pattern of reactivity with the previously reported anti-Mena^11a^ antibody [17] (characterization of the antibody is reported in the Figure S2); anti-Phospho-p44/42 MAPK (Thr202/Tyr204) mouse mAb, p44/42 MAPK rabbit antibody, Phospho-AKT (Ser473) antibody, AKT antibody and Phospho-HER3 were all from Cell Signaling Technology (Beverly, MA); anti-phospho-HER2 (Y1248) from Upstate Biotechnology (Lake Placid, NY); anti-Neu C18, anti-EGFR 1005 and anti-HER3 C17 were from Santa Cruz Biotechnology (Santa Cruz, CA); anti-EGFR (pY1068) phosphospecific antibody obtained from Biosource (Camarillo, CA). After 3 washes of 15 min each, blots were incubated with the appropriate secondary antibody conjugated with HRP for 1 h and then washed again three times. The protein signals were detected by ECL kit (GE-Healthcare). For actin signal, blots were reprobed with 1 µg/ml monoclonal anti-actin, mouse-ascites fluid clone AC-40 (Sigma Aldrich, Poole, UK). X-ray films were scanned by HP Scanjet 5470 and processed by Corel Photo Paint 12.

### RNA extraction and real-time PCR

Five micrograms of total mRNA were extracted using Trizol reagent (Life Technologies Inc, Rockville, MD, USA) to obtain the relative cDNA by first strand cDNA synthesis kit (GE-Healthcare) according to the manufacturer's protocol. Real-time PCRs (RT-PCR) were run on an ABI Prism 7900 RT-PCR machine (Applied Biosystems) using the following cycling conditions: 50°C for 2 min, 95°C for 10 min, and 40 cycles at 95°C for 15 s each followed by termination at 60°C for 1 min. Each sample contained 10 ng template cDNA, 10 µl 2x Taqman Universal Master Mix (Applied Biosystems), 100 nmol/L of each primer, and 200 nmol/L probe in 20 µl volume. Amplification primers and probe (Assay ID Hs00430216) of Taqman gene expression assay (Applied Biosystem) were used for hMena. The *β-Actin* gene used as an endogenous control was amplified using the Taqman gene expression assay (Applied Biosystems). Data from triplicate samples were analyzed with SDS 2.1 software (Applied Biosystems) and relative hMena mRNA expression levels were calculated using the ΔΔ*C*_T_ method.

### Two-Dimensional Electrophoresis (2DE)

Cells were washed extensively with PBS, pellets lyophilized and solubilized with 2DE solubilization buffer (9 M Urea, 10 mM Tris, 4% CHAPS, 65 mM DTT, 2% IPG buffer ampholine pH 3-10, protease inhibitor cocktail). Protein samples (250 µg) were applied to 7 cm IPGstrips pH 3-10NL (GE-Healthcare) by in-gel rehydration. Iso-electro Focusing (IEF) was performed with an IPGphor system (GE-Healthcare) following a standard protocol as described [33]. Strips were equilibrated in 50 mM pH 8.8 Tris-HCl buffer containing 6 M urea, 30% glycerol, 2% SDS and 2% DTT, followed by an incubation in the same buffer replacing DTT with 2.5% iodoacetamide. The strips were loaded on the top of 10% acrylamide SDS-PAGE gels for the second dimension separation. Proteins were electrontransferred onto nitrocellulose membranes and Western blot performed as described above. Images were acquired at high resolution and 2D immunoreactivity patterns analyzed using Progenesis PG240 v2005 software (Nonlinear dynamics, Newcastle, UK). Relative molecular mass (Mr) were estimated by comparison with Mr reference markers (Precision, Bio-Rad, Hercules, CA, USA) and isoelectric point (p*I*) values assigned to detected spots by calibration as described in the GE-Healthcare guidelines.

### Phosphatase treatment

Lambda Protein Phosphatase (λ-PPase) (NewEngland BioLabs, Ipswich, MA, USA) treatment was performed as described [34] with modifications. In brief, pelleted cells (25×10^6^) were lyophilized and resuspended in lysis buffer (1% w/v NP-40, 1% w/v SDS, 50 mM Tris pH 7.6, 150 mM NaCl, protease inhibitor cocktail). Sixty µl of lysate, corresponding to 600 µg of protein, were brought to a final volume of 600 µl with deionized water followed by the addition of 20 µl of 20 mM MnCl_2_ solution and 20 µl of λ-PPase buffer. For each addition, the solution was gently mixed. The mixture was divided into two aliquots, and 300 units of λ-PPase was added to one of the aliquots. After mixing, aliquots were incubated for 6 h at 30°C. Proteins were acetone precipitated at −20°C and used for 2DE analysis.

### Small-interfering RNA treatment

Cells in exponential growth phase were plated in 6-well plates 3×10^5^ cells/well. After 24 h cells were transfected with 100 nmol/L hMena-specific pooled siRNA duplexes (siENA SMART pool) or control non-specific siRNA (Dharmacon, Lafayette, CO) using Lipofectamine 2000 reagent (Invitrogen). After 72 h cells were analyzed by Western blot or, after 24 h, cells were serum starved for 18 h and then treated with 100 ng/ml EGF or 10 ng/ml NRG1 for additional 24 h for Western blot analysis or [^3^H]thymidine incorporation assay.

### [^3^H]Thymidine Incorporation Assay

The day after siRNA transfection cells from each well were transferred to 4 wells of 48-well-plates and treated with growth factors as above described. For ^3^H-thymidine incorporation assay, ^3^H-thymidine (Perkin Elmer Life and Analytical Sciences, Boston, MA) was added at 5 µCi/mL for 4 h on the last day of treatment. Following medium removal, cells were washed twice with cold PBS, treated with 10% trichloroacetic acid for 30 min at 37°C, solubilized with 0.4 N NaOH and counted for incorporation of ^3^H on β liquid scintillation counter in 5 mL of scintillation fluid. Each experiment was done in quadruplicate and results were expressed as the means of at least three separate experiments.

### Patients and Tissue Specimens

hMena was analyzed by immunohistochemistry in a series of 286 breast cancer patients ranging in age 28–86 years (median, 53 years) subjected to breast cancer surgery at the Regina Elena Cancer Institute (Rome, Italy) between 2000 and 2004. Follow-up data were obtained from hospital charts and by correspondence with the referring physicians. As shown in Table 1, the group included 246 (86%) invasive ductal carcinomas, 28 (10%) invasive lobular carcinomas, and 12 (4%) other histotypes. Among these, 171 (60%) were pT1, 80 (29%) pT2 and 28 (11%) pT3/4, 154 (54%) were node negative and 132 (46%) node positive, 27 (10%) G1, 146 (54%) G2 and 96 (34%) G3. Ki67 was found positive in 122 tumors (43%) and negative in 164 (57%), while hMena was overexpressed in 190 (66%) cases and negative in 96 (34%). Tumors were graded according to Bloom and Richardson and staged according to the Unione Internationale Contre le Cancer tumor-node-metastasis system criteria and histologically classified according to the World Health Organization [35]. Moreover, we studied hMena distribution according to the molecular subtypes identified by a few protein biomarkers, namely ERα, PgR, HER2. They were 177 (62%) Luminal A (LA, ERα/PgR positive and HER2 negative), 34 (12%) Luminal B (LB ERα/PgR positive and HER2 positive), 27 (9%) Triple Negatives (TN, ERα/PgR,HER2 negative) and 48 (17%) HER2 subtypes (HS, ERα/PgR negative, HER2 positive).

The study was reviewed and approved by the ethics committee of the Regina Elena National Cancer Institute, and written informed consent was obtained from all patients.

### Immunohistochemistry and CISH

hMena immunostaining was performed by using a monoclonal antibody from BD Transduction (San Jose, CA, USA; 2.5 µg/ml). Anti estrogen (6F11 MoAb) and progesterone (1A6 MoAb) receptors were purchased from Novocastra (Menarini, Florence, Italy); HER-2 (A0485 pAb) and Ki67 (MIB-1 MoAb) antibodies from DAKO (Milan, Italy); phospho-p44/42 MAPK and phospho-AKT antibodies from Cell Signaling (SIAL, Rome, Italy). Immunostaining was revealed by a streptavidin-biotin enhanced immunoperoxidase technique (Super Sensitive MultiLink, Menarini) in an automated autostainer. Diaminobenzidine was used as chromogenic substrate.

The intensity of hMena cytoplasmic staining was scored from 0 to 3 as previously described [15]; Ki67 percentage, based on the median value of our series, was regarded as high if >15% of the cell nuclei were immunostained.

HER-2 immunostaining was performed following the manifacturer's protocol and protein overexpression was determined as defined in the HercepTest kit guide: score 0, 1+, 2+, 3+. All IHC 2+ tumors were analyzed with chromogenic in situ hybridization (CISH) as previously described [36] in order to determine the HER2 gene copy level. For statistical analysis, negative (HER2 IHC score 0, 1+ and 2+ cases lacking gene amplification) and positive (HER2 IHC score 2+ with gene amplification cases and score 3+) groups were created.

P-MAPK was considered positive when >25% of the neoplastic cells showed a distinct cytoplasmic staining. Breast cancers showing a distinct and intense cytoplasmic immunostaining for P-AKT were scored as strongly positive (2+), independent of the percentage of stained cells.

Evaluation of the immunohistochemical results was done independently and in blinded manner by two investigators (M.M., E.M.).

### Statistical analysis

All experiments were repeated a minimum of three times. Data collected from [^3^H]thymidine incorporation assay were expressed as means ± standard deviation (SD). The data presented in some figures are from a representative experiment, which was qualitatively similar in the replicate experiments. Statistical significance was determined by Student's *t* test (two tailed) comparison between two groups of data sets. Asterisks indicate significant differences of experimental groups compared with the corresponding control condition (*P*<0.05, see figure legends). All the analysis were performed using GraphPad Prism 4, V4.03 software (GraphPad Inc., San Diego, CA). Change in the phosphorylation status was evaluated using Progenesis PG240 v.2006 software (Nonlinear dynamics) by optical density indicated as normalized spot volume. Normalization was done multiplying the total spot volume by the constant factor 100 which produces spot percentage volume.

The Chi-Square test and Fisher exact text, when appropriate were used to assess the relationship between hMena positivity and the other biological parameters (HER2, Ki-67, P-AKT, P-MAPK). The Chi-Square test for linear trend was also applied.

The disease-free survival (DFS) curves were estimated by the Kaplan-Meier product-limit method. The log-rank test was used to assess differences between subgroups. Significance was defined at the *p<*0.05 level. Statistical analysis for survival were performed with SPSS statistical software version 11.5 (SPSS inc., Chicago IL, USA).

## Supporting Information

Figure S1 Western Blot analysis of MCF7 and MDA-MB-361 after 72 h transfection with control and hMena/hMena^11a^ -specific siRNAs with pan-hMena antibody. As loading control blots were probed with anti-Actin antibody (1 µg/ml).
(TIF)

Figure S2 **Characterization of anti-hMena^11a^ antibody.**
***A***. Western blot analysis with 1 µg/ml of anti-hMena^11a^ antibody on lysates of SKBr3, expressing hMena^11a^, and BT549, negative for hMena^11a^ expression, transfected with either control vector (pcDNA3) or hMena^11a^.
***B–C.*** Western blot analysis of DAL cells transfected with either control vector (pcDNA3) or hMena^11a^ with 1 µg/ml (B) or 0.1 µg/ml (C) of anti-hMena^11a^ antibody. As loading control blots were probed with anti-Actin antibody (1 µg/ml).
(TIF)
