# Supplementary figures and images for: The Cooperation between hMena Overexpression and HER2 Signalling in Breast Cancer
Source: PLoS One. 2010 Dec 30;5(12):e15852. doi: 10.1371/journal.pone.0015852 (PMC3012725; doi:10.1371/journal.pone.0015852)

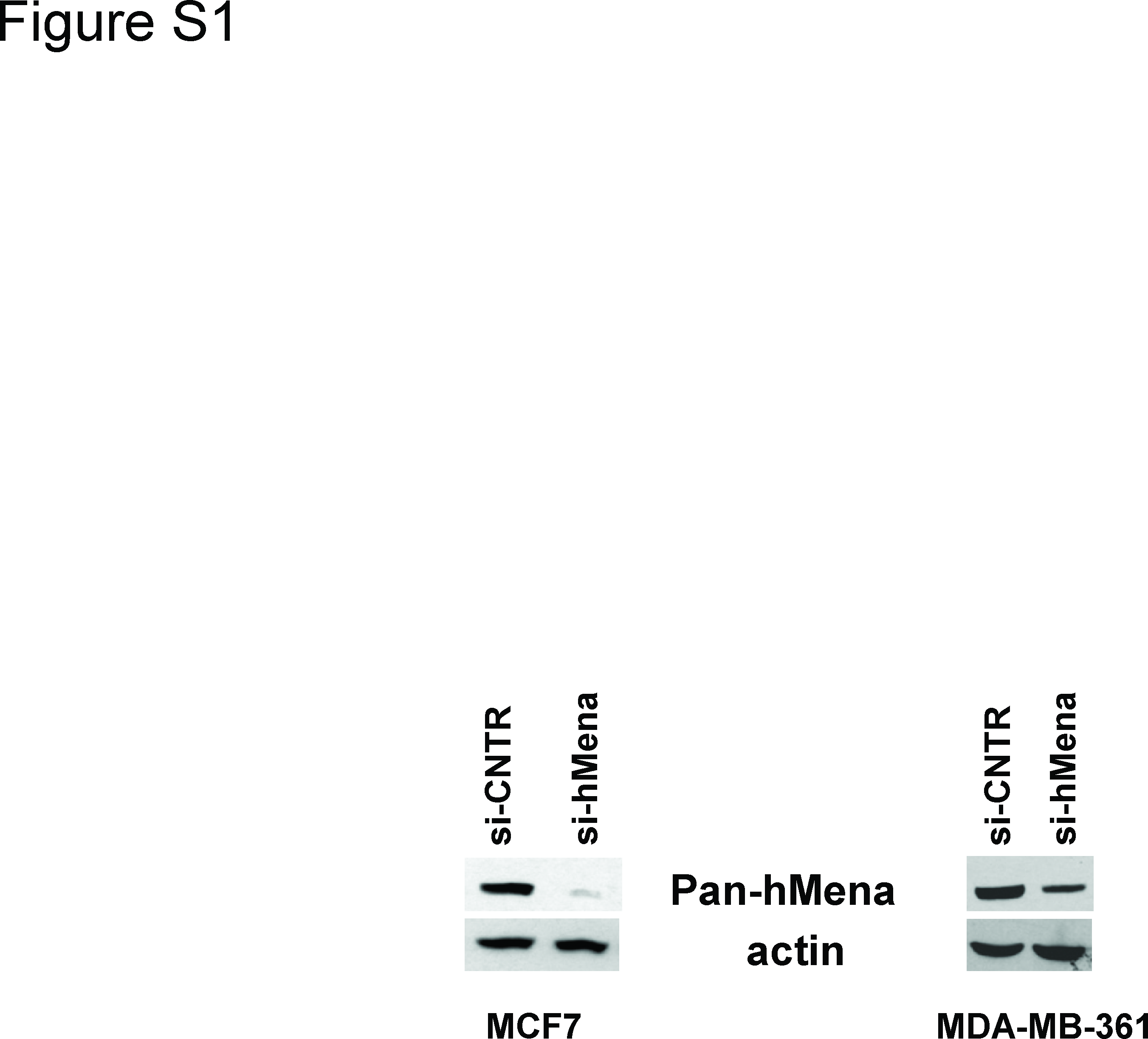

Supplement: Figure S1 — Western Blot analysis of MCF7 and MDA-MB-361 after 72 h transfection with control and hMena/hMena11a -specific siRNAs with pan-hMena antibody. As loading control blots were probed with anti-Actin antibody (1 µg/ml). (TIF) [file pone.0015852.s001.tif]

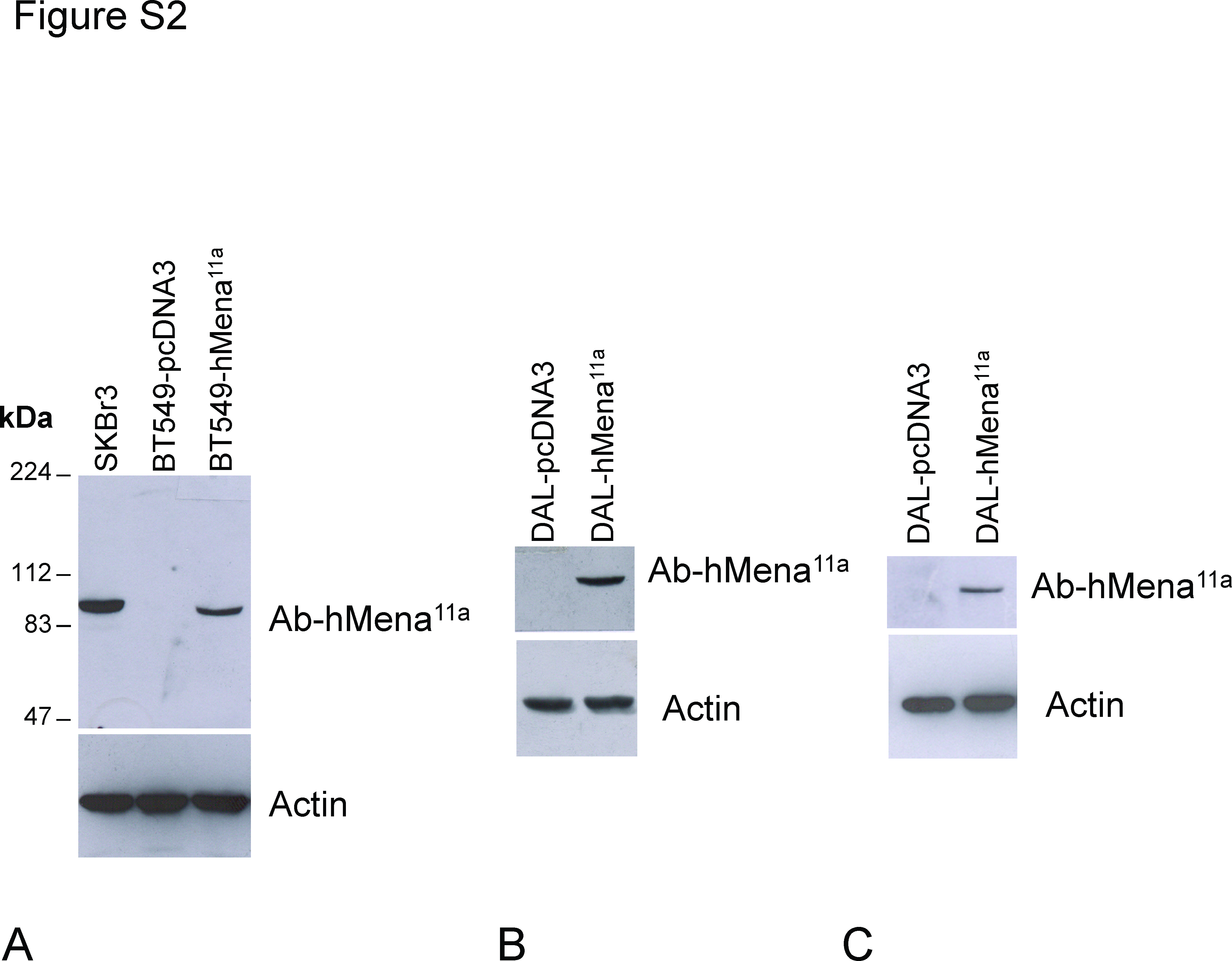

Supplement: Figure S2 — Characterization of anti-hMena11a antibody. A. Western blot analysis with 1 µg/ml of anti-hMena11a antibody on lysates of SKBr3, expressing hMena11a, and BT549, negative for hMena11a expression, transfected with either control vector (pcDNA3) or hMena11a. B–C. Western blot analysis of DAL cells transfected with either control vector (pcDNA3) or hMena11a with 1 µg/ml (B) or 0.1 µg/ml (C) of anti-hMena11a antibody. As loading control blots were probed with anti-Actin antibody (1 µg/ml). (TIF) [file pone.0015852.s002.tif]
